# Mesodiencephalic junction GABAergic inputs are processed separately from motor cortical inputs in the basilar pons

**DOI:** 10.1016/j.isci.2022.104641

**Published:** 2022-06-18

**Authors:** Ayoub J. Khalil, Huibert D. Mansvelder, Laurens Witter

**Affiliations:** 1 Department of Integrative Neurophysiology, Amsterdam Neuroscience, Center for Neurogenomics and Cognitive Research (CNCR), Vrije Universiteit Amsterdam, 1081HV Amsterdam, the Netherlands; 2 Department for Developmental Origins of Disease, Wilhelmina Children’s Hospital and Brain Center, University Medical Center Utrecht, 3584 EA Utrecht, the Netherlands

**Keywords:** Physiology, Neuroscience, Cell biology

## Abstract

The basilar pontine nuclei (bPN) are known to receive excitatory input from the entire neocortex and constitute the main source of mossy fibers to the cerebellum. Various potential inhibitory afferents have been described, but their origin, synaptic plasticity, and network function have remained elusive. Here we identify the mesodiencephalic junction (MDJ) as a prominent source of monosynaptic GABAergic inputs to the bPN. We found no evidence that these inputs converge with motor cortex (M1) inputs at the single neuron or at the local network level. Tracing the inputs to GABAergic MDJ neurons revealed inputs to these neurons from neocortical areas. Additionally, we observed little short-term synaptic facilitation or depression in afferents from the MDJ, enabling MDJ inputs to carry sign-inversed neocortical inputs. Thus, our results show a prominent source of GABAergic inhibition to the bPN that could enrich input to the cerebellar granule cell layer.

## Highlights

• The basilar pontine nuclei (bPN) receive input from the mesodiencephalic junction (MDJ)
• MDJ input to bPN is GABAergic and monosynaptic
• Individual basilar bPN neurons do not integrate inputs from the motor cortex and MDJ
• MDJ inputs to bPN most likely represent recoded cortical inputs

## Graphical abstract

**Figure:**
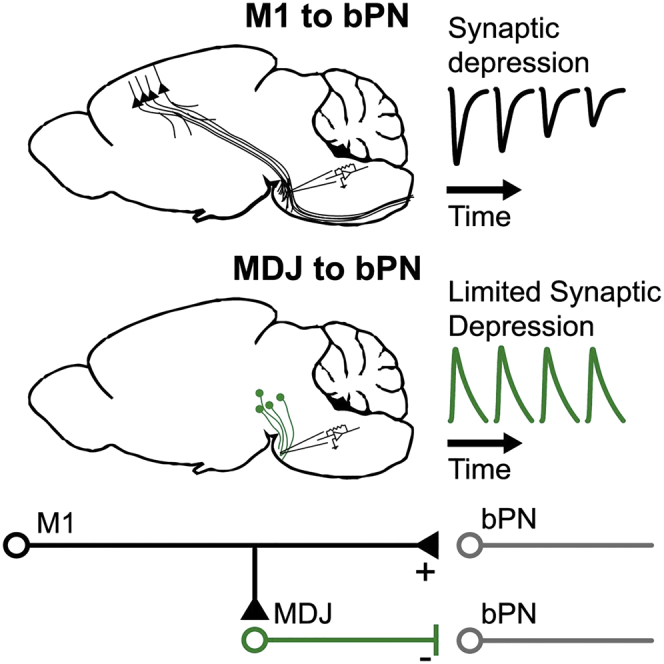

## Introduction

Motor control relies on brain-wide networks. Motor cortex directs voluntary movements (Guo et al., 2015) and the cerebellum coordinates movements (Manto et al., 2012). Reciprocal connections between these structures are necessary for proper motor control. Indeed, the cerebellum projects to the motor cortex via the thalamus (Sawyer et al., 1994; Aumann 2002; Gornati et al., 2018), while the motor cortex projects to the cerebellum via the pontine nuclei (Schwarz and Mock 2001; Kratochwil et al., 2017). This closed-loop connectivity is proposed to enable forward and inverse models for motor control (Wolpert et al., 1998; Shadmehr and Krakauer 2008). Interestingly, other parts of the neocortex and cerebellum are also connected (Kelly and Strick 2003; Henschke and Pakan 2020; Pisano et al., 2021), potentially enabling similar computational mechanisms for cognitive processes (Ito 2008).

This places the pontine nuclei at the nexus of information transfer between neocortex and cerebellum. Indeed, afferents from the basilar pontine nuclei (bPN) constitute the principal source of mossy fibers in the cerebellum (Kratochwil et al., 2017). The bPN also receives inputs from numerous non-neocortical regions of the brain (Burne et al., 1981; Wiesendanger and Wiesendanger 1982; Kosinski et al., 1986; Mihailoff et al., 1988, 1989). These afferents generally terminate in topographically organized zones in the bPN (Leergaard and Bjaalie 2007; Proville et al., 2014; Kratochwil et al., 2017). Similarly, mossy fibers originating from the bPN project to specific zones in the cerebellum (Päällysaho et al., 1991; Mihailoff 1993; Odeh et al., 2005; Huang et al., 2013; Kratochwil et al., 2017). Consequently, the bPN is often considered to be a relay for information destined for the cerebellum rather than having a role in active processing.

Still, synaptic plasticity of inputs to the bPN has been described, suggesting a potential way of input processing (Möck et al., 1997) and shaping spiking activity in the bPN (Schwarz et al., 1997; Möck et al., 2006; Guo et al., 2021). Furthermore, various sources of GABAergic input to bPN neurons have been suggested (Border et al., 1986; Mihailoff and Border 1990; Möck et al., 1999), but these inputs have not been physiologically confirmed or characterized, precluding conclusions about their function and integration in the cerebro-cerebellar circuit.

Here we identify the mesodiencephalic junction (MDJ) as the main source of GABAergic signaling to the bPN. This inhibition does not seem to interact with afferents from the motor cortex at the single neuron or network level, even though their projections overlap in the bPN. In contrast to strongly depressing motor cortex inputs, GABAergic inputs from MDJ show remarkably little short-term depression. Finally, using rabies tracing we show that pontine-projecting MDJ neurons receive prominent neocortical inputs, similar to bPN neurons themselves. These results suggest that the bPN contains separate streams for processing information from neocortex directly and sign-inverted neocortical inputs.

## Results

### The basilar pontine nuclei connect the fore- and midbrain to the cerebellum

**Figure 1:**
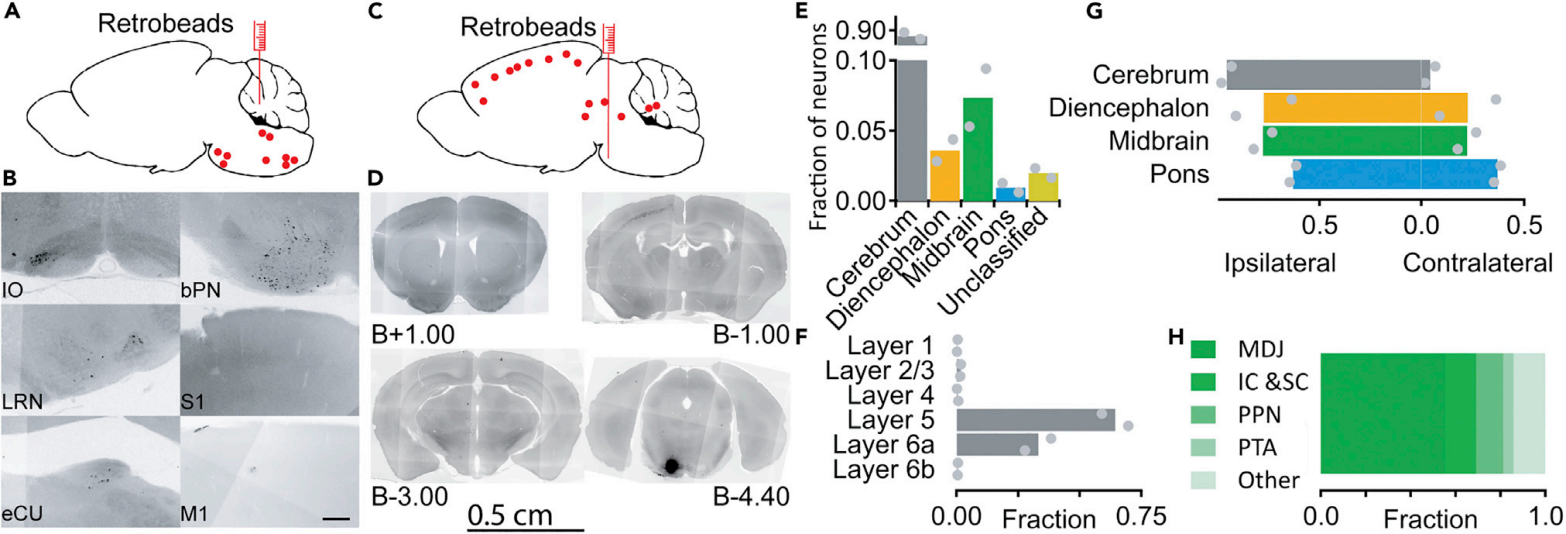
The basilar pontine nuclei are intermediate between the cerebral cortex and the cerebellum (A) Schematic representation of a retrobead injection in cerebellum (N = 2 animals). Retrobeads were injected into the cerebellar nuclei and retrograde labeling was assessed (red dots). (B) Examples of retrobead-labeled neurons in the inferior olivary nucleus (IO), lateral reticular nucleus (LRN), external cuneate nucleus (eCU), basilar pontine nuclei (bPN), and the absence of labeled neurons in the primary sensory cortex (S1), and primary motor cortex (S1). Images were produced by acquiring monochromatic photos in the red spectrum and then inversing these images. As a result, retrobeads are depicted in black. Scale bar represents 50 μm. (C) Same as for A, but for retrobead injections in the bPN (N = 2 animals). (D) Same as for B, but for retrograde labeling after injections in bPN. Example sections are shown with labeling concentrated in the neocortex, sparse signal of retrogradely labeled neurons in midbrain, and dense staining in the injection site. Numbers after capital letter B indicate distance from Bregma. (E) Quantification of retrograde labeling from bPN (see materials & methods). Average (bars) and individual data points (grey dots) are shown. (F) Retrograde labeling in neocortex is predominantly found in deeper layers (layers 5 and 6a). (G) Retrograde labeling is predominantly ipsilateral. (H) Of all midbain inputs MDJ provides the most prominent input to bPN. Each colorized bar indicates the average fraction of labeled neurons in a subdivision of the midbrain. MDJ: mesodiencephalic junction; IC: Inferior colliculus; SC; Superior colliculus; PPN: Pedunculopontine nucleus; PTA: pretectal area.

To characterize the anatomical cortico-cerebellar pathways that run via the pontine nuclei, we first injected retrobeads into the cerebellum (Figure 1). Injections were conducted in the white matter of paravermal lobule 5 (Figure 1A, N = 2) and retrograde labeling was assessed after 14 days. As expected, retrograde labeling from the cerebellum was observed in the inferior olivary nucleus, external cuneate nucleus, lateral reticular nucleus, and bPN (Figure 1B), but not in the cerebral cortex (Figure 1B). We then injected retrobeads into the bPN to investigate afferent regions (Figure 1C, N = 2). Injections were confined to the basilar pons, with minimal invasion of overlying structures (Figure S1). We predominantly observed inputs from the ipsilateral side of the brain (Figure 1G), with 90% of retrogradely labeled neurons localized to deep layers (Figure 1F) of the neocortex (Figure 1E). The midbrain was the most prominent source of subcortical afferents (5% in total, Figure 1H), followed by thalamus and hypothalamus (3%, Figure 1E). This confirms that the bPN is a prominent intermediary between the cerebrum and the cerebellum.

### Monosynaptic inputs from M1 to the basilar pontine nuclei display marked synaptic depression

**Figure 2:**
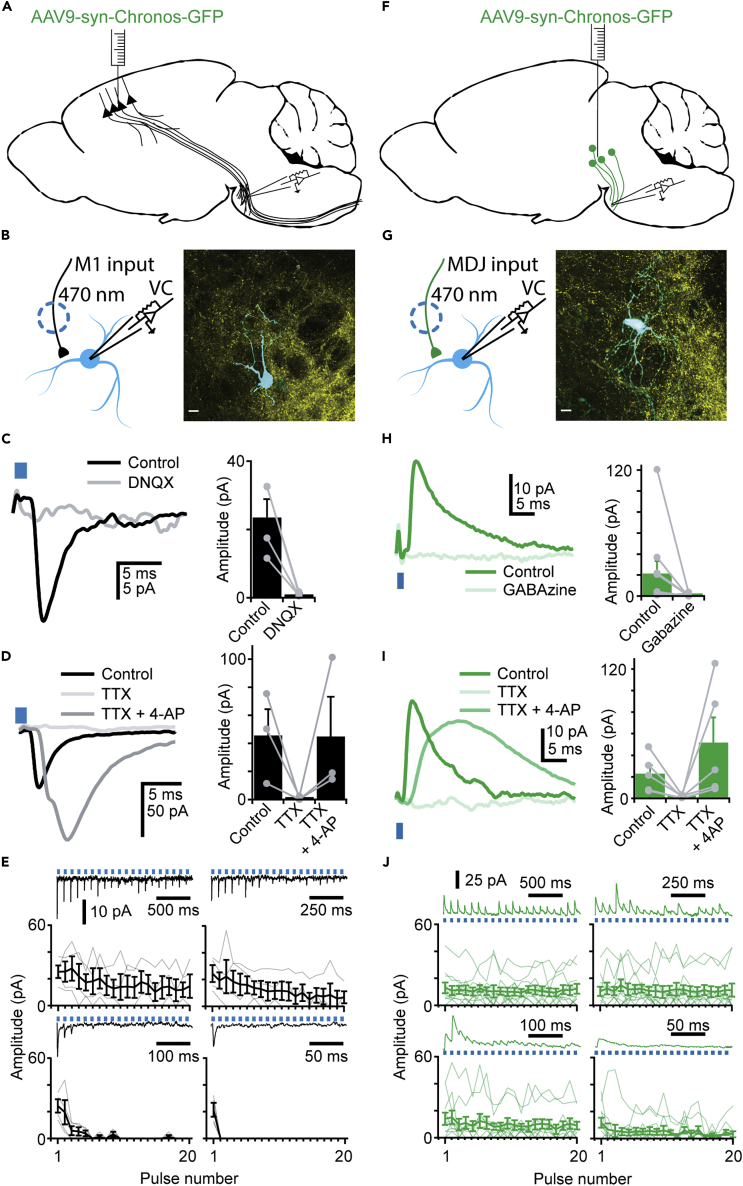
Optogenetic stimulation of M1 and MDJ afferents to the bPN (A) Schematic overview of AAV virus injection in M1. (B) Schematic overview of the experimental patch-clamp approach (left). Axons from channelrhodopsin-expressing pyramidal neurons in motor cortex are present in bPN, and can be stimulated with short, intense pulses of light. Putative postsynaptic neurons were recorded in voltage clamp at −70 mV. (right) Post-hoc recovered and stained neuron (cyan) showing GFP+ M1 fibers (Yellow) in close proximity to each other. Scale bar represents 10 μm. (C) M1 inputs are effectively blocked with DNQX (n = 4). Example traces (left) and quantification of peak amplitude (right). (D) M1 makes monosynaptic contacts to bPN (n = 3). Example traces (left) and quantification of peak amplitudes (right). (E) M1 inputs undergo profound synaptic depression depending on the stimulus frequency (n = 4). Average input amplitude is shown per pulse number. Grey lines represent data from one cell, average shown in black. Example traces are shown for each frequency (starting from the top left graph in clockwise direction: 10, 20, 100, 50 Hz). (F) Same as for A but for virus injection in MDJ. (G) Same as for B, but for neurons receiving input from MDJ: Schematic overview of experimental approach (left). Axons from channelrhodopsin-expressing neurons in MDJ are present in bPN, and can be stimulated with short, intense pulses of light. Putative postsynaptic neurons were recorded in voltage clamp at 0 mV (right). A recovered neuron is shown (cyan) with GFP + MDJ fibers (Yellow) in close proximity. Scale bar represents 10 μm. (H) MDJ inputs are effectively blocked with Gabazine (n = 10). Example traces (left) and quantification of peak amplitudes (right). (I) MDJ makes monosynaptic contacts to bPN (n = 5). Example traces (left) and quantification of peak amplitudes (right). (J) MDJ inputs show limited short-term depression at all frequencies (N = 11, organized as in E). Data are represented as mean ± SEM.

To characterize the short-term plasticity of cortico-pontine synapses, we expressed Chronos in the motor cortex (M1) to enable the visualization and stimulation of M1 afferents to the bPN. Whole-cell voltage-clamp recordings from bPN neurons at −70 mV were made, and M1 axons were stimulated with short pulses of blue light (Figure 2A and 2B). All inputs evoked from M1 had a short delay to onset (2.4 ± 0.06 ms), a fast-rising phase and decay (10-90%: 1.0 ± 0.22 ms and 90-10%: 20 ± 12 ms, respectively), and were reduced to 4.4 ± 0.8% of the original response by DNQX application (ACSF: 20 ± 5 pA; DNQX: 1.0 ± 0.3 pA; p < 0.001; n = 4 neurons; Figure 2C), confirming that M1 provides glutamatergic inputs to bPN. During train stimulation, we observed prominent short-term synaptic depression of M1 inputs across all tested frequencies, with more pronounced depression at higher frequencies and later in the stimulus train (Figure 2E, n = 4 neurons in N = 4 mice). To check for possible opsin-specific influences on these inputs, we repeated these experiments with ChrimsonR. ChrimsonR-evoked responses were more depressed at higher stimulation frequencies (Figure S2A), which is likely owing to incomplete recovery of the ion channel (Klapoetke et al., 2014). Therefore, we assessed all short-term plasticity with the stimulation of Chronos. In mice expressing Chronos in M1, synaptic responses recorded in the bPN were depressed at 50 and 100 Hz after a 20-pulse train stimulus to 0.7 ± 0% and 0 ± 0% of initial amplitude, respectively (steady-state, average of last five responses). After train stimuli at 10 and 20 Hz, responses were depressed to 41 ± 3% and 22 ± 2%, respectively. To confirm that M1 inputs to the bPN are monosynaptic, we applied tetrodotoxin (TTX) to block AP-generated neurotransmitter release. In this situation, stimulated axons can only depolarize during the optogenetic stimulation, after which they are quickly repolarized, preventing invasion of positive charge into synaptic boutons. As expected, optogenetically evoked responses were virtually absent in the presence of TTX (2.2 ± 0.2% of the original response; ACSF: 50 ± 18 pA; TTX: 1.0 ± 0.7 pA; n = 3). Subsequent co-application of 4-aminopyridine (4-AP), which prolongs optogenetically evoked depolarization and therefore increases the likelihood of stimulation of boutons, even with remote axonal stimulation, recovered the synaptic responses (130 ± 56% of amplitude in ACSF; TTX + 4-AP: 50 ± 28 pA, n = 3; Figure 2D; Repeated measures ANOVA on measurements normalized to ACSF condition: F(2,2) = 11.21, p = 0.02; Post-hoc Bonferroni corrected t-tests ACSF vs TTX p < 0.001; TTX vs TTX+4AP p = 0.22; ACSF vs TTX+4AP p = 0.98). These results show that M1 provides prominent, but strongly depressing monosynaptic glutamatergic inputs to bPN neurons.

### The mesodiencephalic junction sends prominent monosynaptic GABAergic inputs to the basilar pontine nuclei

Our retrograde tracing experiments (Figure 1) showed that in addition to neocortical regions, some subcortical brain regions also provide inputs to the bPN. These regions might provide GABAergic inputs as has been suggested before (Border et al., 1986; Mihailoff et al., 1988, 1989; Mihailoff and Border 1990; Mihailoff 1995). To investigate possible GABAergic signaling in the bPN, we stained sections of mouse brain for the enzyme Glutamate decarboxylate 67 (GAD67) to identify GABAergic neurons. We never observed GAD67+ somata in the bPN of these mice, but we did observe prominent and numerous GAD67+ boutons (Figures S2B and S2C; N = 6 mice). We further confirmed these observations in GAD-GFP mice (Chattopadhyaya et al., 2004) (Figure S2D; N = 4 mice). This indicates that there is a prominent extrinsic source of GABA in the bPN. Closer investigation of the afferent areas to bPN revealed that the majority of inputs from midbrain arose from the MDJ (3% of all projections to the bPN, Figure 1H), an area intimately involved with the cerebellar circuit (Ruigrok 2004). Glutamatergic MDJ neurons that project to the inferior olive have been described previously (de Zeeuw et al., 1989; Ruigrok and Voogd 1995) and these neurons are positioned intermixed with neurons that contain other neurotransmitters (De Zeeuw and Ruigrok 1994).

To confirm that the MDJ is a source of GABAergic inputs to the bPN, we injected AAVs to express Chronos in this region. In acute slices, we performed whole-cell recordings in regions of the bPN that also receive inputs from M1. We observed outward currents in neurons clamped at 0 mV, with a short rise, and long decay (2.1 ± 0.36 ms and 140 ± 46 ms, respectively) when stimulating with light (Figures 2F and 2G). These inputs were reduced to 6 ± 7% in the presence of Gabazine (ACSF: 20 pA ± 11 pA; Gabazine: 0.5 ± 0.41 pA; n = 10 neurons; p < 0.001; Figure 2H). Furthermore, we did not observe a change in holding current (ACSF: 140 ± 28 pA vs Gabazine: 160 ± 33 pA, n = 10 neurons, p = 0.27), indicating that inhibition from MDJ to bPN neurons is predominantly phasic

Contrary to glutamatergic M1 inputs, GABAergic MDJ inputs showed remarkably little short-term synaptic plasticity at intervals >20 ms, even after a 20 pulse stimulation-train, we observed 108 ± 5% and 105 ± 7% of the initial amplitude for 10 and 20 Hz stimulation trains, respectively (Figure 2J, n = 11 neurons in N = 9 mice). The amplitude of responses was only depressed toward the end of a pulse train at frequencies ≧50 Hz (to 55 ± 7% and 14 ± 7% for 50 and 100 Hz stimulus trains, respectively). Similar to the observed ChrimsonR effects on M1 inputs, MDJ afferents expressing ChrimsonR showed enhanced short-term synaptic depression (Figure S2A). To confirm that inputs from the MDJ are monosynaptic, we applied TTX followed by the combined application of TTX and 4-AP. Inputs from the MDJ are blocked upon TTX application (5 ± 3.9% of the response in ACSF; ACSF: 23 ± 8 pA vs TTX: 1.5 ± 0.61 pA; n = 5 neurons) and subsequently rescued after co-application with 4-AP (to 400 ± 760% of ACSF response; TTX +4-AP: 50 ± 23pA; n = 5 neurons Figure 2I; Repeated measures ANOVA on measurements normalized to ASCF condition: F(2,4) = 27.77, p < 0.001; Post-hoc Bonferroni corrected t-tests ACSF vs TTX p < 0.001; TTX vs TTX+4AP p = 0.05; ACSF vs TTX+4AP p = 1.00).

### Segregated information streams to the basilar pontine nuclei

**Figure 3:**
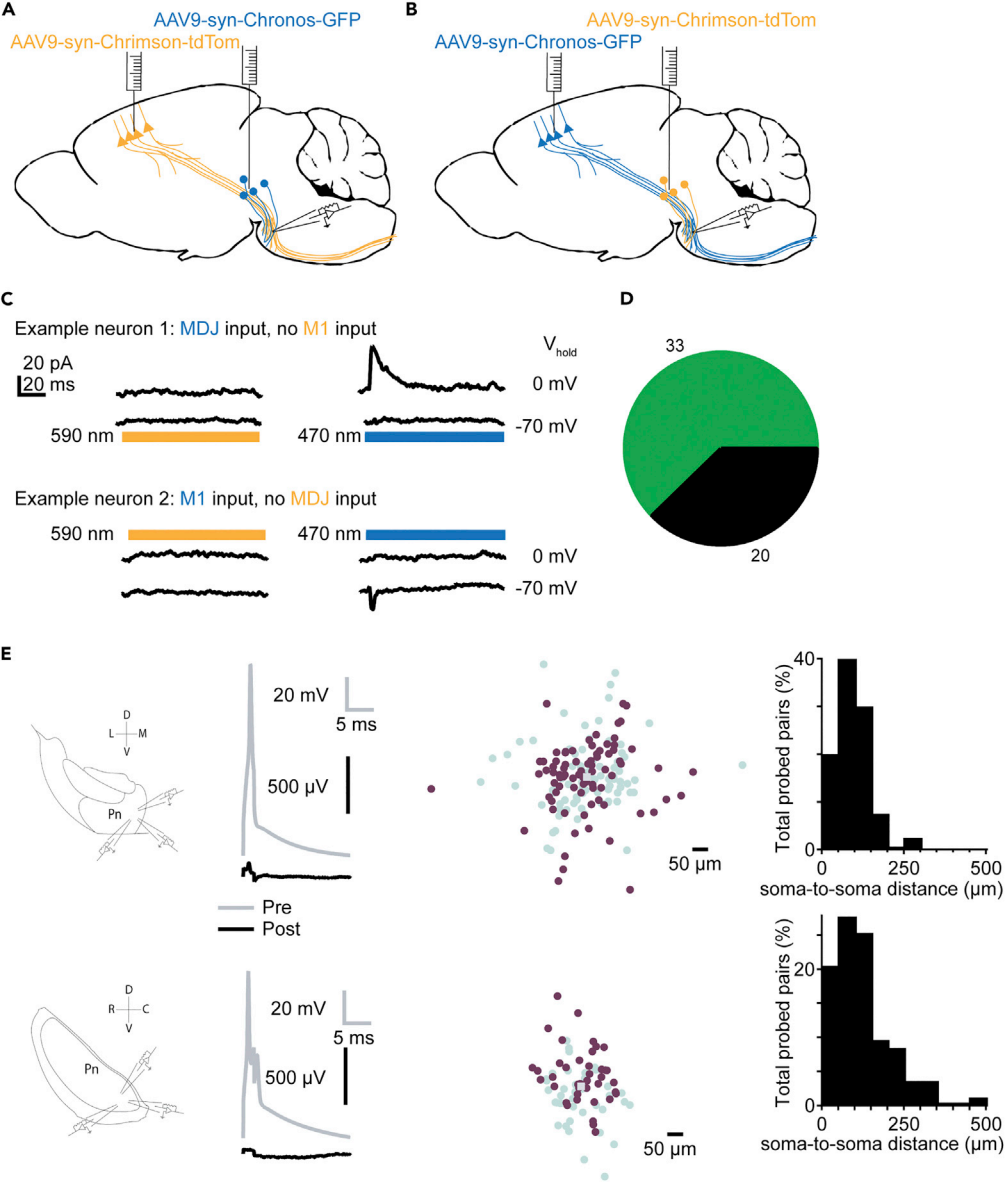
MDJ and M1 inputs likely remain separated information streams in bPN (A) Schematic overview of AAV virus injection in MDJ (Chronos) and M1 (ChrimsonR), or inverse (B). (C) Single neurons receive input from M1, or from MDJ, but not from both. Shown are two typical examples of neurons responsive only to the stimulation of one wavelength, and thus only responsive to one input. (D) Totals of neurons responding to M1 and MDJ stimulation. (E) Experimental setup whole-cell patch-clamp recordings in coronal (n = 168, top) and sagittal (n = 82, bottom) sections of bPN with corresponding example paired recordings of bPN neurons. Presynaptic neurons were depolarized to fire a spike as indicated in grey, responses from postsynaptic neurons are shown in black (held in current clamp at a membrane potential of approximately −70 mV). No postsynaptic responses could be identified following presynaptic spikes. Right: Distances of probed connections measured between pre-synaptic neuron (grey square, middle) and post-synaptic neuron (purple). Teal markers indicate reciprocal distances and are point-mirrored to purple markers. Data from the scatterplot are summarized in a histogram on the far right. Abbreviations: D: Dorsal; V: Ventral; L: Lateral; M: Medial.

Our results thus far indicate that neurons in the bPN receive depressing excitatory input from M1 and inhibitory input from the MDJ that undergoes very little short-term plasticity. A possible role of the bPN is modulating incoming cortical inputs, for example via inhibitory inputs from MDJ. However, this can only be achieved if these inputs interact in a network. To investigate whether single bPN neurons receive inputs from both M1 and from MDJ, we analyzed data from long full-field optical stimulation of all neurons that responded to either M1 or MDJ axon stimulation (see method details). The success rate of evoking opsin-induced currents in bPN neurons was generally low, while we could detect spontaneous excitatory and inhibitory events in many neurons. This indicates that potentially many other sources of input to bPN neurons exist. Neurons were clamped at −70 mV and subsequently at 0 mV to enable the detection of EPSCs and IPSCs, respectively (Figures 3A–3C). Of all bPN neurons that responded to optogenetic stimulation, 60% (33 out of 53) of neurons only received inputs from MDJ and 40% (20 out of 53) only received inputs from M1 Figure 3D). We did not observe any neurons that received both M1 and MDJ inputs, suggesting that these afferents target different neurons within the bPN. To investigate whether these neurons might represent different classes, we compared several passive electrical properties between the two groups. However, we found no statistically significant differences in membrane resistance (M1: 320 ± 49 MΩ; MDJ: 220 ± 25 MΩ; p = 0.08; n = 52), membrane capacitance (M1: 100 ± 15 pF; MDJ: 108 ± 8.2; p = 0.86; n = 52) or membrane decay time constant (M1: 1.18 ± 0.08 ms; MDJ: 1.2 ± 0.10 ms; p = 0.96; n = 52) between these two groups, providing no indication that these neurons represent separate classes (Figure S3). Thus, our results show that convergence of inputs from M1 and MDJ in the bPN is rare if not absent, making it unlikely that MDJ inputs directly modulate M1 inputs in bPN.

Nonetheless, it is possible that inputs from M1 and MDJ indirectly interact in the bPN via a local network. One study reports no short-range interactions between bPN neurons, but this dataset only comprised twenty tested pairs and strictly probed proximate connections with a maximal distance well below 100 μm (Möck et al., 2006). To confirm and expand on this finding, we probed a total of 250 unidirectional connections spaced up to 500 μm apart, in slices cut in both the coronal (n = 168) and sagittal (n = 82) orientation to avoid confounding effects of slice orientation (Shinoda et al., 1992) (Figure 3E). We did not detect evidence of any synaptic contacts between neurons. Therefore, it is also unlikely that M1 and MDJ inputs interact via a local circuit, but rather that M1 and MDJ inputs are processed separately by the bPN.

This could explain a recent report that the firing rates of distinct pontine neuron populations differentially change during a voluntary reaching and grabbing task (Guo et al., 2021). If bPN-projecting MDJ neurons are indeed recruited during movement, we predict that they receive prominent inputs from the neocortex.

### Basilar pontine nuclei-projecting mesodiencephalic junction neurons receive input from neocortex

**Figure 4:**
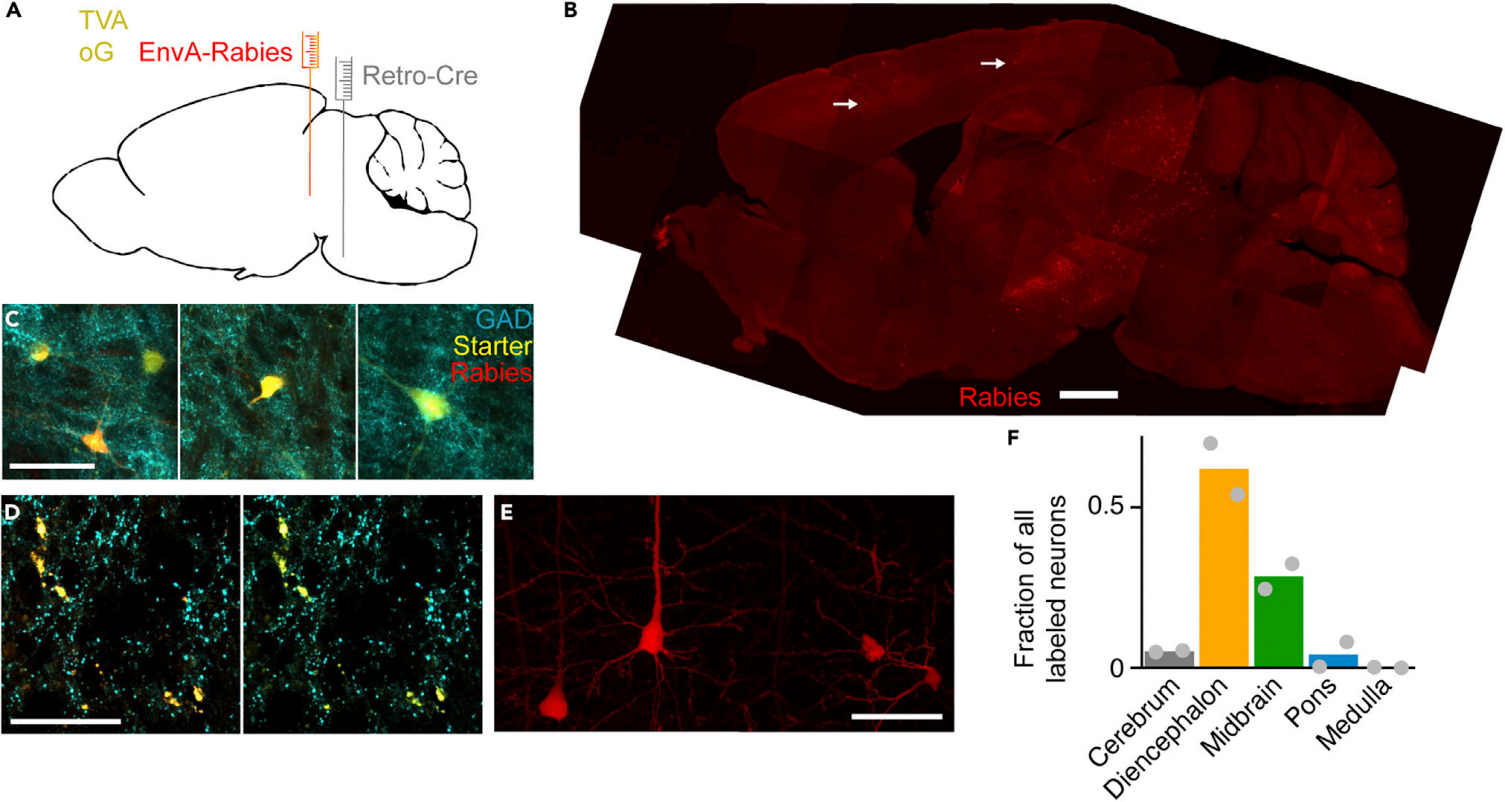
Rabies tracing of inputs to bPN-projecting MDJ neurons (A) Schematic representation of virus injections. AAV2-retro to express cre was injected in the bPN to infect all afferents. At the same time, AAVs to express optimized G-protein and a TVA receptor-GFP construct were injected into the MDJ to enable monosynaptic rabies tracing. Finally, envelop-A rabies virus was injected into the MDJ to label presynaptic partners of neurons in the MDJ that project to the bPN. (B) Low-magnification overview of Rabies+ neurons in a single parasagittal section. White arrows indicate labeled L5 pyramidal neurons. (C) Starter neurons in the MDJ that project to the bPN, containing TVA and optimized G-protein were GFP (yellow), and rabies-virus (red) positive. Post-hoc GAD67-staining (cyan) confirmed the GABAergic nature of these neurons. (D) Presence of GAD+ terminals (cyan) from MDJ starter neurons (yellow) in bPN. Left: GAD, Starter and Rabies. Right: GAD and starter labeling only are shown. (E) Rabies tracing revealed prominent deep layer labeling in neocortex (red). (F) Quantification of two rabies-injected animals as in A showing a consistent strong labeling of diencephalic and mesencephalic structures, and less numerous labeled neurons in cerebrum, pons, and medulla. Scale bar in B represents 1 mm, and 50 μm in C,D & E.

To investigate whether the neocortex projects to bPN-projecting MDJ neurons we used monosynaptic rabies tracing (Wickersham et al., 2006). We first checked whether we could trace connections from neocortex, through bPN to cerebellum. We injected a retrograde AAV (Tervo et al., 2016) into cerebellum to express cre in bPN neurons, followed by AAVs to express TVA and rabies glycoprotein in bPN and subsequent glycoprotein-deleted EnvA rabies virus after one week (see method details). With this approach, we could visualize rabies-infected neurons in neocortex, and GAD+ rabies-infected neurons in MDJ (Figure S4). In other mice, we injected retrograde AAV in the bPN to express Cre in all afferent areas to bPN. Subsequent injections with AAVs to express TVA and optimized G protein were made into the MDJ, followed by pseudotyped rabies virus. In these experiments (Figure 4A, n = 3 mice) we observed widespread labeling of rabies virus throughout the brain (Figures 4B and 4F). As expected, starter neurons in the MDJ were GAD+ (Figure 4C), and we could observe many GAD+ axon terminals in bPN from these neurons (Figure 4D). This confirmed that MDJ GAD+ neurons, indeed, make contacts in the bPN. In the neocortex of these mice, we observed rabies-virus labeled pyramidal neurons in the deep layers of neocortex (Figures 4B and 4E). These results show that GABAergic neurons in the MDJ that project to the bPN receive inputs from neocortex.

## Discussion

We show that the bPN receives prominent inputs from neocortex and from MDJ. Using whole-cell recordings and optogenetic stimulation we show that the bPN receive synaptically depressing glutamatergic inputs from M1 and GABAergic inputs from MDJ that show remarkably little short-term plasticity. Furthermore, we did not observe convergence of M1 and MDJ inputs onto single bPN neurons, and our paired recording data show that convergence via locally connected bPN neurons is exceedingly rare at best. This suggests that M1 and MDJ represent separate streams of information through the bPN. Finally, using Rabies-virus tracing we show that MDJ neurons that project to the bPN receive prominent input from neocortical output neurons. Thus, our results show and characterize a previously unknown source of GABA to bPN from the MDJ, which could provide sign-inversed inputs from neocortex to cerebellar granule cells.

It has long been unclear whether inhibitory inputs to the bPN exist and from where their afferents arise. Several sources of GABAergic inputs have been suggested, including the zona incerta, anterior pretectal nucleus, and cerebellar nuclei (Border et al., 1986). Local interneurons in the bPN have also been suggested to provide GABAergic inhibition (Border and Mihailoff 1985, 1990; Brodal et al., 1988). We did not find a pronounced number of afferents from the sources suggested previously. Instead, retrograde tracing from the bPN produced labeling in the caudal part of the MDJ, which we confirmed to be a source of GABAergic inputs to bPN neurons. The MDJ is a region located in the tegmentum that receives prominent inputs from neocortex and richly innervates the inferior olive with glutamatergic afferents (De Zeeuw et al., 1998; Kubo et al., 2018; Wang et al., 2022). In a functional study, it was suggested that the inhibition of bPN neurons could be induced via a polysynaptic pathway from motor cortex (Guo et al., 2021), and inhibition in bPN neurons has been observed *in vitro* after the stimulation of the cerebral peduncle and the tegmentum (Möck et al., 1997). GABAergic inhibition to bPN neurons, therefore, seems to be completely extrinsic. However, there may still be sources other than the MDJ that provide inhibition to the bPN. For example, in this study, we did not consider possible glycinergic afferents (Aas and Brodal 1990). At the same time, several midbrain regions have been suggested to also provide inputs to bPN neurons, though it remains unclear which neurotransmitters are involved (Mihailoff et al., 1989). Interestingly, we did not observe excitatory inputs during the optical stimulation of MDJ afferents in our experiments, suggesting that MDJ inputs to bPN are exclusively GABAergic.

The bPN are thought to integrate incoming motor and sensory information from the neocortex at the single-cell level (Potter et al., 1978). Indeed, some neurons in the bPN respond only to movement, whereas others are responsive to multiple modalities such as movement and cue (Guo et al., 2021), though this might also reflect earlier integration in the cortex. The precise extent of convergent streams in the bPN remains an important unanswered question. Based on anatomical tracing data, it is suggested that excitatory afferents from different regions could converge onto single bPN neurons despite the general topographical organization (Mihailoff et al., 1988; Lee and Mihailoff 1990; Schwarz and Thier 1999; Leergaard 2003; Leergaard and Bjaalie 2007). It is, therefore, striking that we did not find any convergence of excitatory M1 and inhibitory MDJ inputs in the bPN. Furthermore, we have found no evidence of synaptic connectivity between bPN neurons. Although we cannot unequivocally rule out the presence of synaptically connected bPN neurons, based on our results and a similar earlier report we expect that such connections would be too sparse to be functionally relevant (see also Möck et al., 2006). Thus, information carried by MDJ and M1 inputs very likely remains segregated in the output of the bPN.

In addition to targeting different populations, we show that GABAergic MDJ and glutamatergic M1 inputs are also markedly different in their short-term plasticity. M1 inputs show clear synaptic depression across all tested frequencies, which is particularly prominent during stimulation at relatively high frequencies. Conversely, MDJ inputs undergo little synaptic plasticity except for slight depression towards the end of a pulse train at higher stimulation frequencies. These differences are important, as synaptic plasticity plays an important role in shaping the activity of neurons (Silver 2010). Layer 5 neurons provide the output from neocortex to bPN (Tervo et al., 2016), and respond with changes in firing rate up to 50 Hz during movement (Park et al., 2021; Guo et al., 2021). Our electrophysiological data show that M1 inputs below 50 Hz undergo limited short-term depression, and thus can be reliably transmitted to bPN neurons. Indeed, during reaching, bPN neurons show modulations of their firing rates in line with activity in layer 5 of neocortex (Guo et al., 2021). We took care to estimate short-term synaptic plasticity by stimulating over axons with short pulses of light, while avoiding stimulating over boutons or somata (Jackman et al., 2014). However, even these estimates of short-term plasticity can be confounded by the choice of opsins, generating artificial depression. Indeed, ChrimsonR, a channelrhodopsin with relatively slow kinetics, showed more pronounced depression than Chronos, a faster variant (Klapoetke et al., 2014), indicating that synaptic plasticity estimates are also depending on the speed of the opsin. Still, stimulation with ChrimsonR and Chronos yielded comparable results for frequencies up to 20 Hz, indicating that our estimates of synaptic plasticity probably are not an artifact from optogenetic stimulation in this frequency range. However, we do notice subtle synaptic depression of MDJ inputs in the 50-100 Hz range, which is possibly an optogenetic stimulation artifact. Thus, we cannot exclude the possibility that the short-term plasticity of GABA signaling from the MDJ is frequency dependent.

The mechanism by which inhibition in the bPN contributes to voluntary motor control remains an important question to be addressed. Although our findings do not decisively point to one single mechanism, we are able to rule out several hypotheses. Our rabies tracings suggest that GABAergic MDJ afferents to the bPN could be recruited by cortical activation. This possibly explains why optogenetic stimulation of the motor cortex induces diverse changes in the firing rate of bPN neurons (Guo et al., 2021). Although the effects of inhibition were previously studied by optogenetically silencing the entire bPN (Wagner et al., 2019), our data show that GABAergic MDJ inputs specifically target bPN neurons that likely receive excitatory inputs from cortical areas other than M1. Therefore, we consider it unlikely that feedforward inhibition from the MDJ serves as a gating mechanism (Crowley et al., 2009; Geborek et al., 2013). Furthermore, the phasic nature of GABA signaling in the bPN suggests a timing-dependent mechanism rather than gain adjustment (Silver 2010). It is, therefore, more likely that the MDJ specifically provides the bPN with a negative signal based on neocortex inputs to MDJ. This is further supported by the fact that we only observe purely GABAergic inputs coming from the MDJ. In this arrangement, the bPN would transmit one direct positive signal based on corticopontine inputs, and one negative signal based on cortico-MDJ-pontine inputs. This would greatly enrich the inputs that are provided to the input layer of the cerebellar cortex, which would support cerebellar learning (Chabrol et al., 2015; Cayco-Gajic et al., 2017; Straub et al., 2020).

Thus, we propose that the bPN are more than a passive relay for information destined for the cerebellum. The seemingly targeted projection of GABAergic MDJ afferents suggests that inhibition to the bPN fulfills a specific role that is likely timed with cortical activation. An important remaining question is how the activation of the MDJ impacts voluntary motor behavior and whether its timing differentially affects the outcome of a planned movement. Further investigations of inhibition in the bPN should focus on performance during a learned voluntary behavioral task in order to address these questions. Given its crucial position within the cerebro-cerebellar circuit, expanding our knowledge of bPN functionality will likely aid in further understanding the mechanisms underlying voluntary motor control and cerebellar learning.

### Limitations of the study

In the present study, we used viral tracing and optogenetics as tools to study M1 and MDJ inputs to the bPN, as well as their synaptic plasticity and possible convergence onto single neurons. Even though channelrhodopsin is a widely used light-gated ion channel, the kinetics of channelrhodopsin is not shared across variants, which may differentially affect estimations of synaptic plasticity. We based our conclusions on input data recorded using Chronos. Despite having the fastest on-off kinetics among all available variants, we here confirm that Chronos-induced axonal stimulation is still only reliable up to firing frequencies of around 50 Hz. This precludes us from accurately describing synaptic plasticity of M1 and MDJ inputs during high frequency (i.e. >50 Hz) stimulation. Still, owing to the known properties of Chronos and the comparable results between Chronos and ChrimsonR, the estimates of synaptic plasticity for both M1 and MDJ inputs in the lower frequency range (10-20 Hz) are likely to be accurate.

To investigate whether M1 and MDJ inputs converge at the single-cell level we analyzed responses to full-field light stimulation. Although in none of the neurons recorded we observed responses to both M1 and MDJ afferent stimulation, it is possible that such neurons do exist and can be found through sampling a much larger number of neurons.

We hypothesized that the inhibition of M1 inputs in the bPN could have important functional implications. However, if we missed convergence of M1 and MDJ afferents onto single bPN neurons as a result of undersampling, we still expect that such convergence is unlikely to hold substantial functional significance owing to its rarity.

We then investigated whether there are local networks in the bPN by simultaneously patching pairs of neurons. We performed these experiments in brain slices *in vitro*. It is important to note that many dendrites and axons are cut during the slicing procedure, particularly at this slice thickness (i.e. 250 μm), thus lowering the chance of finding synaptically connected neurons. We tried to minimize this issue by sampling from pairs in two slicing orientations. However, owing to this limitation, we cannot rule out the possibility of a few local connections in the bPN. Ideally, the presence of local networks in the bPN is studied *in vivo* where the complete bPN network remains intact.

Finally, the use of rabies viral tracing has taken a flight over the past few years, but several shortcomings have been reported, such as an undersampling of presynaptic partners, selective tropism, and heavy dependence on helper plasmid and injection timing (Ginger et al., 2013). In our current study, the use of glycoprotein-deleted rabies tracing could have biased our results to a particular neuronal population, potentially missing another population of neurons presynaptic to bPN neurons. At the same time, the viral tracing results might underestimate the total number of neurons impinging on bPN neurons.

## STAR★Methods

### Key resources table

**Table:** 

REAGENT or RESOURCE	SOURCE	IDENTIFIER
**Antibodies**		

Alexa Fluor 647 secondary antibody (Goat anti mouse)	Invitrogen	A21235
Mouse monoclonal anti-GAD67 (clone 1G10.2)	Merck-Millipore	MAB5406
Streptavidin Alexa 647	Invitrogen	S32357

**Bacterial and virus strains**		

AAV2r-hSyn1-chI-iCre-WPRE-SV40	University of Zurich viral vector facility	N/A
rAAVdj-hsyn1-dlox-TVA-2A-EGFP-2a-oG(rev)-dlox-WPRE-bGhp(A)	Gift from K.K. Conzelmann	N/A
Rabies-SAD-dG-tdTomato	Gift from K.K. Conzelmann	N/A
syn.ChrimsonR-tdTomato.WPRE.bGH	(Klapoetke et al., 2014)	Addgene AAV9; 59171-AAV9
syn.Chronos-GFP.WPRE.bGH	(Klapoetke et al., 2014)	Addgene AAV9; 59170-AAV9

**Chemicals, peptides, and recombinant proteins**		

4-Aminopyridine (4-AP)	Sigma-Aldrich	275875
6,7-dinitroquinoxaline-2,3-dione (DNQX)	Hello Bio	HB0261
Biocytin	Molekula	36219518
Hydrobromide (Gabazine)	Hello Bio	HB0901
Tetrodotoxin citrate (TTX)	Hello Bio	HB1035

**Experimental models: Organisms/strains**		

C57BL/6J mouse	Charles River	N/A
GAD67-GFP C57BL/6 mouse	(Chattopadhyaya et al., 2004)	N/A

**Software and algorithms**		

CellCounter	Max Planck Institute for Brain Research	https://github.molgen.mpg.de/MPIBR/CellCounter
WholeBrain	(Fürth et al., 2018)	https://github.com/tractatus/wholebrain

### Resource availability

#### Lead contact

Further information and requests for resources and reagents should be directed to and will be fulfilled by Dr. Laurens Witter (l.witter-2@umcutrecht.nl).

#### Materials availability

This study did not generate new unique reagents.

### Experimental model and subject details

#### Animals

Male and female wt C57BL/6J mice were used for acute slice experiments. Mice were housed socially (max. four per cage) and had *ad libitum* access to chow and water. All experimental procedures were approved by the Central Authority for Scientific Procedures on Animals and local animal welfare body of the VU University and VU University Medical Center (Amsterdam, Netherlands) and carried out in accordance with European and Dutch law.

### Method details

#### Intracranial virus and tracer injections

Microinjection needles were pulled from 3.5″ borosilicate glass capillaries (Drummond SCI, USA) on a Sutter P-87 puller (Sutter, CA) and backfilled with mineral oil before virus solution was loaded. AAV9 viruses were purchased from Addgene (USA) syn.Chronos-GFP.WPRE.bGH and syn.ChrimsonR-tdTomato.WPRE.bGH were injected at 4⋅10^12^ vg/mL titer and 1.5⋅10^12^ vg/mL respectively. Retrograde AAV2 virus was purchased from University of Zurich vector core. AAV2r-hSyn1-chI-iCre-WPRE-SV40 was injected at a titer of 7.9⋅10^12^ vg/mL. Rabies virus (Rabies-SAD-dG-tdTomato) and AAV helper virus (rAAVdj-hsyn1-dlox-TVA-2A-EGFP-2a-oG(rev)-dlox-WPRE-bGhp(A)) were a generous gift from Klaus Conzelmann. All mice used for optogenetic experiments received intracranial virus injections at postnatal 21. For all surgeries, mice received Carprofen (5 mg/kg s.c.) and Buprenorphine (50 μg/kg s.c.) pre-operatively. A second Carprofen injection (5 mg/kg s.c.) was administered 24 h post-surgery. Mice were kept under general anesthesia during surgery with Isoflurane (0.5–1%). Ear bars were placed to secure the skull, a small amount of Lidocaine cream was applied before placement. Local analgesia was applied by injecting a small volume of Lidocaine (2%) underneath the scalp before incising the skin. The scalp was cut and folded open to expose the skull, holes were drilled to access the injection sites, and virus was delivered via injection. (relative to bregma (Paxinos and Watson 1998) (in mm), M1: AP 1.30; ML 1.08L; DV 1.20, MDJ: AP -3.50; ML 0.50L; DV 3.00 Cerebellum: AP -6.2; ML 1.5R; DV 2.0 bPN: AP-4.0 ML 0.5L DV 5.5). For optogenetic experiments, total volume of 500 nL was injected per site in steps of 50 nL/min using a Nanoject II (Drummond SCI, USA) set to the ‘slow’ rate (23 nL/s). The microinjection needle was left in place for 5 min before and after injection. Mice were sacrificed for acute slice experiments at least two weeks after viral injection to allow for adequate expression. For tracing experiments, total volumes between 10 and 100 nL were injected per site and needles were left in place for 15 min before retraction. Retrobead transport was assessed after 14 days. For rabies tracing mice were injected with AAV to express cre, oG and TVA in one surgery. After 1 week rabies virus was injected, after which we waited another week before mice were perfused with 4% formaldehyde solution in 0.1 M phosphate-buffered saline (PBS) for analysis.

#### Acute slice preparation

Acute slices were prepared for optogenetic experiments (sagittal orientation) and paired recordings (sagittal or coronal). Before decapitation, mice first received a lethal pentobarbital injection (120 mg/kg i.p.) and were perfused with ice cold N-Methyl- D -glucamine (NMDG) solution containing (in mM): NMDG 93, KCl 2.5, NaH_2_PO_4_ 1.2, NaHCO_3_ 30, HEPES 20, Glucose 25, sodium pyruvate 3, sodium ascorbate 5, MgSO_4_ 10, CaCl_2_ 0.5, adjusted to 315 mOsm ± 5 and pH 7.3. After decapitation, the brain was removed from the skull and sliced in the same oxygenated ice-cold NMDG solution. Brains were sliced using a ceramic blade (Campden Instruments ltd., England) and slices (250 μm) were collected in an oxygen-perfused brain slice chamber filled with a holding solution containing (in mM): NaCl 92, KCl 2.5, NaH_2_PO_4_ 1.2, NaHCO_3_ 30, HEPES 20, Glucose 25, sodium pyruvate 3, sodium ascorbate 5, MgSO_4_ 10, CaCl_2_ 0.5, adjusted to 305 ± 5 mOsm. Slices were kept oxygenated at room temperature until the moment of recording.

#### Acute slice whole-cell recordings

During all acute slice experiments, whole-cell recordings were acquired at a temperature of 33 ± 1 ^⁰^C. Brain slices were placed in a bath continuously perfused with oxygenated ACSF containing (in mM): NaCl 125, KCl 2.5, NaH_2_PO_4_ 1.25, NaHCO_3_ 26, Glucose 25, MgCl_2_ 1, CaCl_2_ 1.3, adjusted to 305 ± 5 mOsm. Borosilicate glass capillaries were pulled to produce patch-pipettes with a resistance of 3–6 MΩ. For optogenetic experiments, patch-pipettes were filled with a cesium methanesulfonate-based pipette solution containing (in mM): CsMethanesulfonate 115, TEA 25, HEPES 10, EGTA 0.2, QX-314 Cl 5, NaCl 4, MgATP 2, Na_3_GTP 0.4, Na_2_Phosphocreatine 10. For paired patch-clamp experiments, patch-pipettes were filled with a potassium gluconate-based solution containing (in mM): KGluconate 135, KOH 31, NaCl 10, HEPES 10, EGTA 10, Na_2_ATP 4, Na_3_GTP 0.4. Both internal solutions were adjusted to pH 7.2 and 310 mOsm. Biocytin (0.05%) was added to internal solution on the day of the experiment. Cells were loaded with biocytin during whole-cell patch clamp recordings and resealed at the end of the experiment. Slices were then transferred to paraformaldehyde (PFA, 4%) and fixed for at least 48 h. Passive membrane properties were calculated from the average response to three test pulses (10 ms, 10 mV) given in whole-cell voltage clamp prior to the experiment.

#### Optogenetic stimulation

Optogenetic responses were evoked using a 4-channel LED system (DC4100 & LED4D114; Thorlabs inc., USA). Cells were voltage clamped at −70 and 0 mV and screened for responses using full field 100 ms optical stimulation at all four wavelengths (405, 470, 505 & 590 nm). When responses (EPSCs or IPSCs) were observed, the light source was restricted to a small beam (±100 μm diameter) with high intensity (>100 mW/mm^2^) >500 μm away from the soma of the recorded neuron to allow reliable axonal stimulation of afferents and minimize potential activation of polysynaptic pathways (Jackman et al., 2014). Optical stimulation was delivered in trains of twenty pulses with a 10 s intertrain interval, and was alternated per sweep in a pseudorandom order (20–100 – 50–10 Hz). All input characterizations are based on afferents expressing Chronos. A short negative voltage (50 ms, −10 mV) was injected at the start of each sweep to monitor access resistance throughout the experiment. Voltage clamp recordings were acquired at a 50.0 kHz sample rate with a 10 kHz low pass filter. Cells from optogenetic experiments were analyzed on the following conditions: (1) optical stimulation at 470 or 590 nm evoked a response at −70 mV or 0 mV holding potential; (2) at least nine sweeps per frequency were collected. Responses following stimulation were defined as optogenetically evoked inputs if they exceeded the threshold set at 2σ of the baseline. Responses that did not reach the computed threshold were not considered in the analysis. Response amplitudes were computed on averaged sweeps. The peak amplitude was detected within an eight millisecond time window after each light pulse. Then, the response was determined by calculating the average maximum amplitude over a 1-ms time window of the peak amplitude. Baseline was defined as the average amplitude over a 2-ms time window before optic stimulation.

#### Paired whole-cell recordings

Sagittal or coronal slices were prepared for paired recordings. Up to three neurons were recorded at the same time, and potential connections between neurons were probed by evoking spike trains successively in each neuron. Ten action potentials were evoked presynaptically using current injections of 2 nA at 50 Hz, followed by a single current injection after 500 ms. Cells were kept at or around resting membrane potential throughout recording to detect EPCSs. Current clamp recordings were acquired at a 50.0 kHz sample rate with a 10 kHz low pass filter. We did not compensate for the liquid junction potential.

Cells from paired whole-cell patch clamp experiments were analyzed when: (1) stimulation evoked action potentials (APs); (2) cells did not have a negative leak current exceeding 500 pA; (3) recordings had a stable resting membrane potential; (4) at least fifteen sweeps were collected. To detect connections, we looked for EPSCs in the average postsynaptic response in the first 18 ms after the AP to accommodate for mono- and disynaptic connections. Then, the postsynaptic response was determined by calculating the average amplitude over a 1-ms time window of the peak amplitude.

#### Pharmacology

Gabazine (10 μM) was bath-applied to inhibit postsynaptic GABA_A_ responses. AMPA and kainate receptors were inhibited with 6,7-dinitroquinoxaline-2,3-dione (DNQX, 10 μM). TTX (1 μM) was applied to inhibit voltage-gated sodium channels. Voltage-gated potassium channels were inhibited with 4-Aminopyridine (4-AP, 100 μM). All antagonists were bath-applied and perfused at least 5 min before the start of a recording.

#### Histology

For neurons recorded *in vitro*, slices were first washed in phosphate-buffered saline (PBS, 0.1 M, 3 × 15′) containing (in mM): NaCl 137, KCl 2.7, NaH_2_PO_4_ 12, KH_2_PO_4_ 1.8. Then, slices were permeabilized in Triton-X (PBS-T, 0.5%; 2 h). Following permeabilization, slices were again washed in PBS (3 × 15′) and stained with Streptavidin-Alexa 647 (1:500 in 0.5% PBS-T). Finally, slices were washed in PBS (4 × 15′) and embedded in Mowiol (2%) on glass microscope slides for confocal imaging. Tracer-injected brains were washed in 0.1 M PBS and embedded in 11% gelatin for whole-brain sectioning. 50–100 μm sections were made on a Leica VS1000 vibratome and collected directly to glass slides (retrobead tracing, coronal slices), or in 3 jars per side (sagittal slices, 6 jars total per brain). Staining for GAD67 was performed on one jar per side of the brain, yielding 24–30 sections for analysis. Sections were washed 3 × 5′ in PBS with 0.025% Triton X-, blocked for 30’ (PBS+0.025%TX and 5% normal Donkey Serum), and subsequently incubated with GAD67 antibody O/N (1:500 MAB5406, Merck-Millipore) in PBS. The next day sections were washed 3 × 5′ in PBS, incubated for 2 h with Alexa647 secondary (Goat anti mouse, A21235, ThermoFisher), again washed 3 × 5′ in PBS and mounted on slides with mowiol (2%). Cells recovered from *in vitro* recordings, and sections from rabies tracing experiments were imaged on a confocal microscope (Nikon), retrobead tracing was visualized on an epifluorescence microscope (Zeiss).

#### Quantification of labeled neurons

Retrobead tracing was analyzed by first marking all labeled neurons by hand via cellcounter in matlab (https://github.molgen.mpg.de/MPIBR/CellCounter) and then aligning the sections with labeled neurons using the wholebrain tool in R (Fürth et al., 2018) to the Allen Brain Atlas between bregma +3.0 and −5.6. For rabies tracing, sections were visualized under a Zeiss Axio Observer Z1, photographed and counted by hand.

### Quantification and statistical analysis

All statistical analyses were performed in Igor Pro version 7. To compare the effects of pharmacological blockers on synaptic input, one sample t-tests were performed on data normalized to ACSF. For TTX and TTX+4AP experiments, repeated measures ANOVA was used on measurements normalized to the ACSF condition. Post-hoc testing to establish different groups was done using the paired t-test. p values less than 0.05 were considered statistically significant.

## Data Availability

• All data reported in this paper will be shared by the lead contact upon request. • This paper does not report original code. • Any additional information required to reanalyze the data reported in this paper is available from the lead contact upon request.
