# Supplementary material for: Mesodiencephalic junction GABAergic inputs are processed separately from motor cortical inputs in the basilar pons
Source: iScience. 2022 Jun 18;25(7):104641. doi: 10.1016/j.isci.2022.104641 (PMC9254490; doi:10.1016/j.isci.2022.104641)
Supplement: Document S1. Figures S1–S4 [file mmc1.pdf]

iScience, Volume *25*

Supplemental information

Mesodiencephalic junction GABAergic inputs are processed separately from motor cortical inputs in the basilar pons

Ayoub J. Khalil, Huibert D. Mansvelder, and Laurens Witter

Injection 1

Supplementary Figure 1

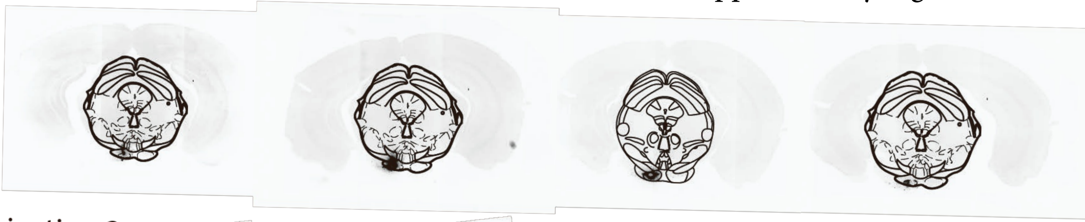

Injection 2

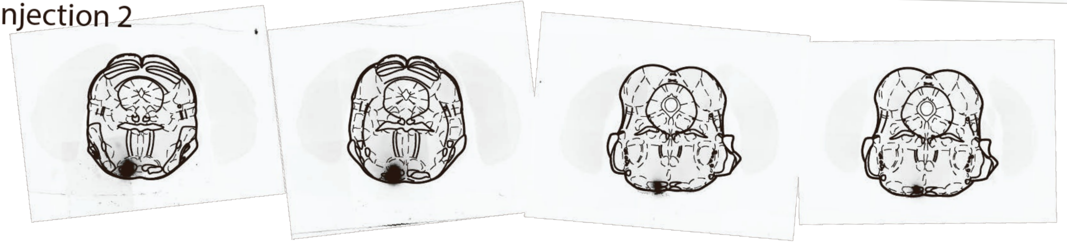

Supplementary Figure 2

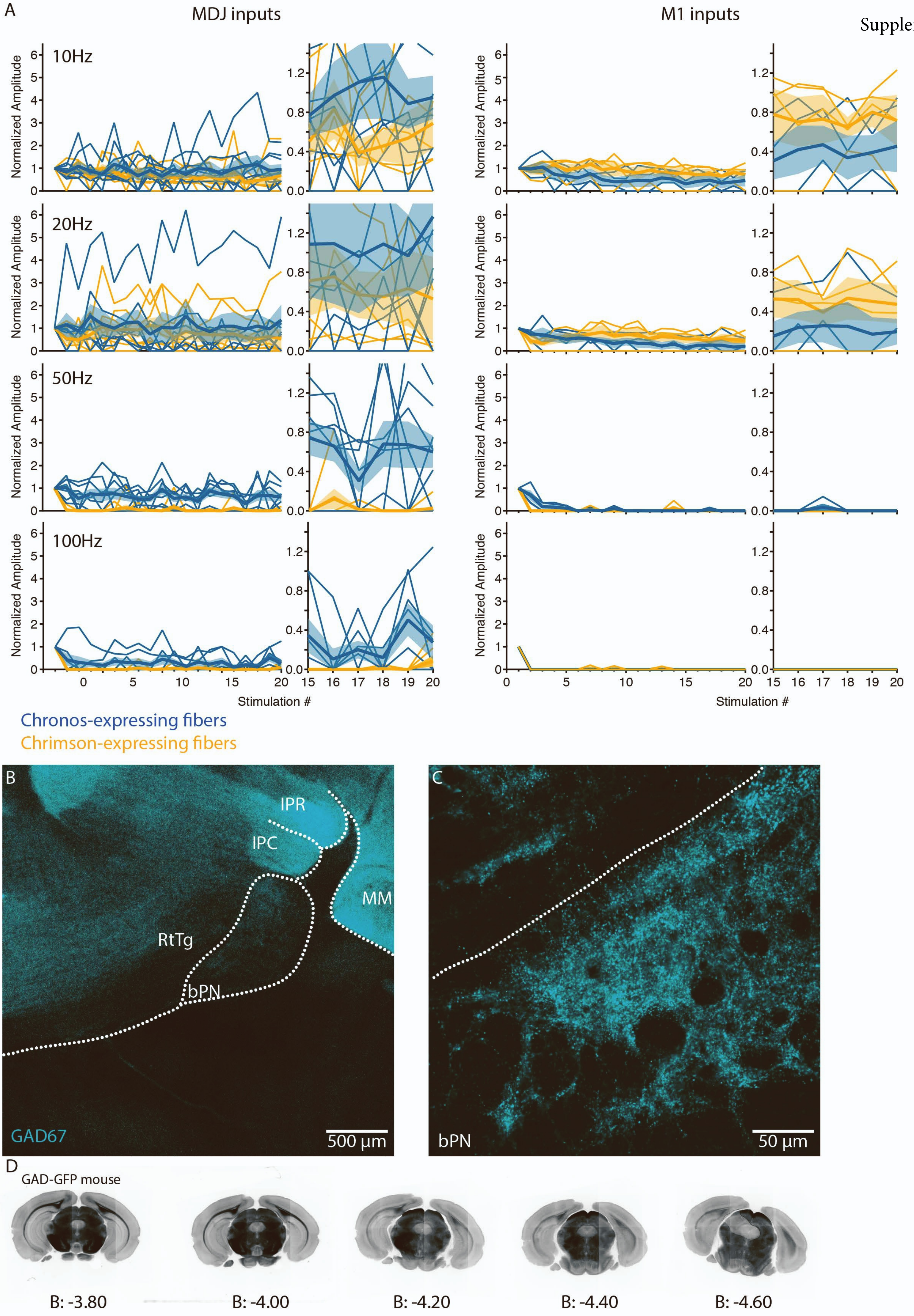

Supplementary Figure 3

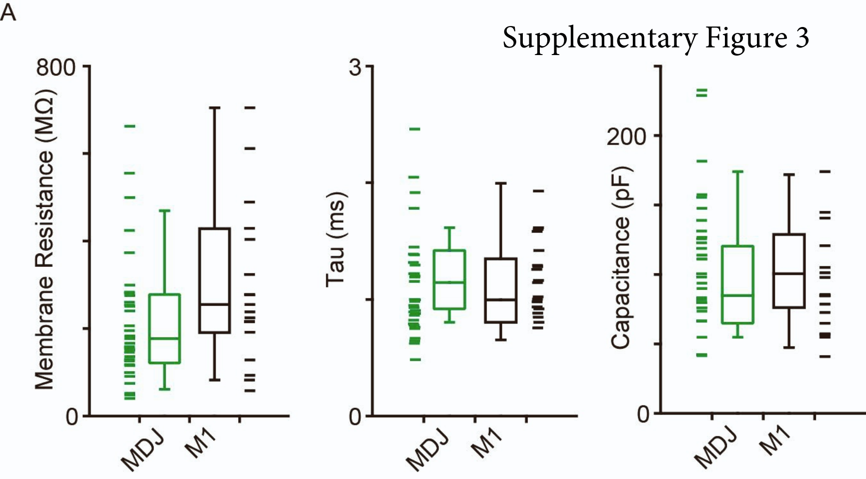

Supplementary Figure 4

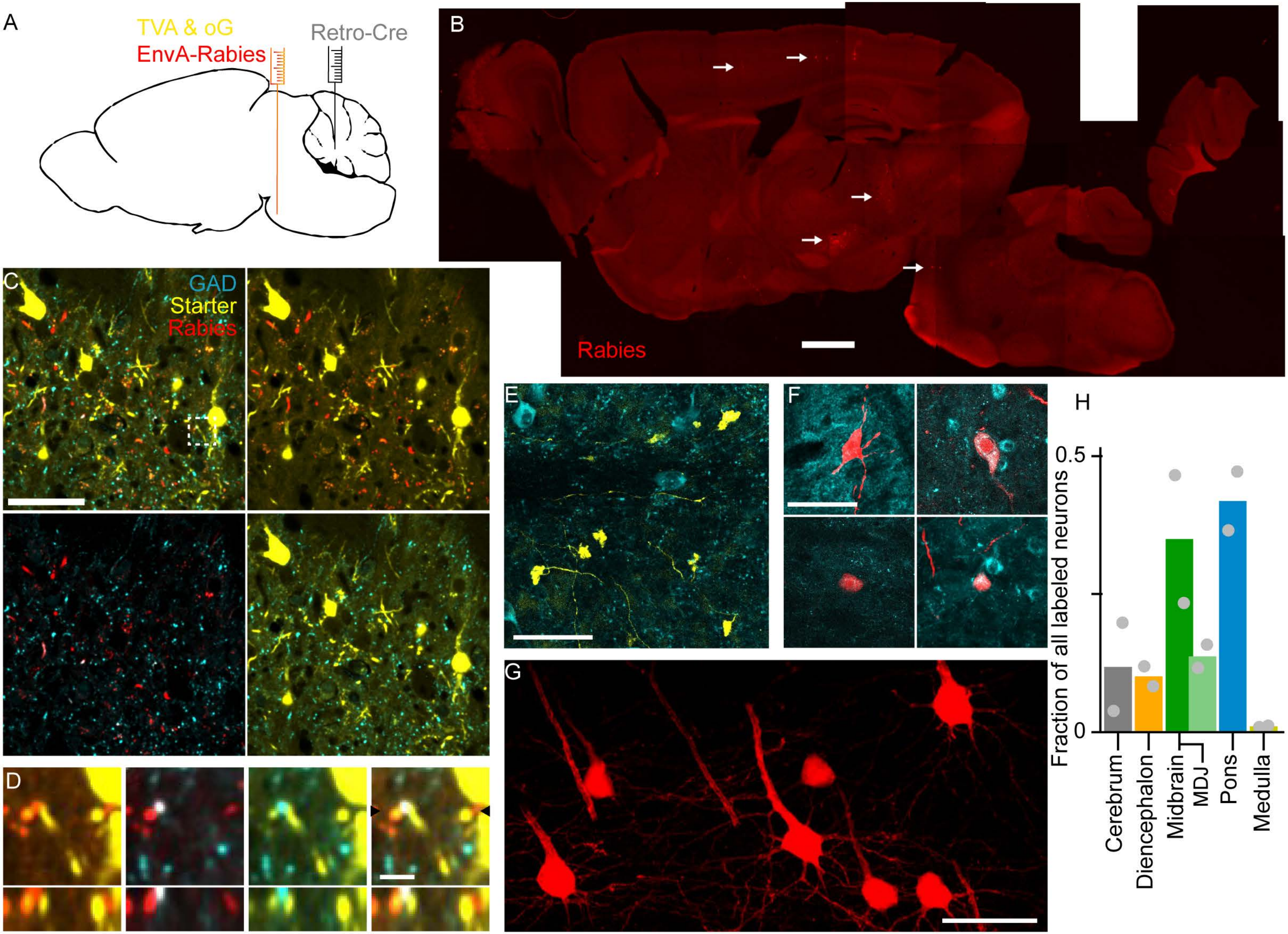

Supplementary figures

Supplementary figure 1: Injection locations for retrobead tracing from bPN, Related to Figure 1.

Two mice were injected with retrobeads in their bPN (top and bottom). Tracer injection locations are shown with the atlas superimposed.

Supplementary figure 2: Optogenetic stimulation of ChrimsonR and Chronos and overview of GABAergic neurons and projections, Related to Figure 2.

(A) MDJ inputs (left) were stimulated via Chronos (blue) or ChrimsonR (Orange) in separate experiments. When stimulating with ChrimsonR more pronounced synaptic depression can be observed compared with Chronos. Especially at higher frequencies (>50Hz) and at the end of the train (second column) this is more pronounced. M1 inputs (right) were stimulated in a similar manner via Chronos and ChrimsonR, resulting in comparable results. (B) GAD67 staining of sections of mice show that there are no GABAergic neurons in the bPN of mice. Compare bPN with areas with known prominent GABAergic neurons (IPR, IPC, MM). RtTg: Reticulotegmental nucleus of the pons; IPC: Caudal subnucleus of the Interpeduncular Nucleus; IPR: Rostral subnucleus of the Interpeduncular Nucleus; MM: Medial Mammillary Nucleus. (C) Magnification of bPN showing prominent GABAergic fibers. (D) GAD-GFP mice show that there is absence of GABAergic neurons in bPN, but GABAergic fibers can be distinguished.

Supplementary Figure 3: Electrophysiological characterization of passive membrane properties of MDJ and M1-receiving bPN neurons, Related to Figure 3.

(A) No differences found in membrane resistance, membrane time-constant and capacitance between groups of MDJ and M1 input-receiving neurons. Horizontal lines represent single neurons, box plot shows median, 25th and 75th percentile. Bars represent 10th and 90th percentile.

Supplementary Figure 4: Rabies tracing of inputs to bPN, Related to Figure 4.

(A) Schematic representation of injection sites for AAV-Cre (grey), AAVs with TVA and glycoprotein (yellow) and EnvA-Rabies (red). (B) Overview of one sagittal setion showing labeling from Rabies virus throughout the brain. For emphasis, labeling of layer 5 pyramidal neurons, and labeling in diencephalon and midbrain is indicated with white arrows. Scale bar represents 1 mm. (C) In bPN, starter neurons (Yellow) were found together with afferents to bPN (Red). GAD67 staining (Cyan) indicates that some afferents, but none of the starter neurons were GABAergic. (D) Enlargement of the area shown with a white box in (C) indicating overlap between GAD staining and some afferents, but none of the starter neurons. A summed stack (left column) through with orthogonal view (right column) at the location indicated with the black arrowheads in the bottom stack is shown. Scale bar represents 5 µm. (E) Mossy fibers from bPN starter neurons were found in cerebellar cortex,. Scale bar represents 50 µm. (F) In MDJ, GAD67 and rabies positive neurons were observed. (G) Prominent labeling of deep layer pyramidal neurons after injection of rabies virus in bPN, scale bar represents 50 µm. (H) Quantification of the fraction of the total number of rabies-labeled neurons per brain area. Short survival times probably reduced the number of neurons labeled in cortex, emphasizing labeling in midbrain and pons. Labeled neurons in the MDJ represented 14% of all labeled neurons, and 40% of labeled neurons in midbrain.
